# Determination of Cortisol Levels in a Small Volume of Saliva of COVID-19-Recovering Patients During Treatment with Psychotropic Drugs

**DOI:** 10.3390/biomedicines13030697

**Published:** 2025-03-12

**Authors:** Ewelina Dziurkowska, Grażyna Guz-Rzeniecka, Maciej Dziurkowski

**Affiliations:** 1 Department of Analytical Chemistry, Medical University of Gdansk, Gen. J. Hallera 107, 80-416 Gdansk, Poland; 2 Hospital for Nervous and Mental Diseases, Skarszewska 7, 83-200 Starogard Gdanski, Poland; grazyna.guz75@wp.pl (G.G.-R.); mdart2002@yahoo.com (M.D.)

**Keywords:** cortisol, saliva, post-COVID-19-recovered patients, psychotropic drugs, LC-DAD, SPE

## Abstract

**Background/Objectives**: Cortisol levels are increased in stressful situations but can also result from a history of COVID-19 infection. Long-term exposure to high cortisol levels has a destructive effect on the CNS (Central Nervous System) and can lead to depression, among other things. The most commonly used psychotropic drugs reduce cortisol concentrations. **Methods:** The aim of our study was to develop an analytical method to determine the level of the hormone in a small volume of saliva (200 µL) in COVID-19 patients using CNS-active drugs. Solid-phase extraction was used to isolate the analyte, and the determination was performed by liquid chromatography with a diode array detector (LC with DAD). **Results**: The developed method was validated. Its linearity was determined to be in the range of 4–500 ng/mL (R^2^ > 0.9986) and the intra- and inter-day precision expressed as coefficient of variation (CV%) did not exceed 12%. The method was then applied to determine cortisol levels in the saliva of post-COVID-19-recovered patients and healthy volunteers. The determined cortisol levels were 12.24 ± 7.33 ng/mL in the recovered patients and 4.11 ± 1.46 ng/mL in the healthy subjects, respectively. A comparison of the results showed that cortisol levels in the recovered patients and healthy volunteers were significantly different statistically. **Conclusions**: The developed method allowed for the determination of cortisol in a small volume of saliva. Comparison of cortisol concentration in healthy individuals and COVID-19 recoveries indicates that the hormone level in both groups significantly differed statistically, and the psychotropic drugs used did not reduce cortisol concentration in COVID-19 patients. The results obtained indicate that the psychotropic drugs used did not reduce cortisol concentrations in COVID-19 patients.

## 1. Introduction

Cortisol, secreted by the adrenal cortex, is also known as the stress hormone. Cortisol release follows a diurnal rhythm and is controlled directly by the adrenocorticotropic hormone (ACTH) and indirectly by the corticotropin-releasing hormone (CRH). Under physiological conditions, healthy adults, in stress-free situations, secrete between 10 and 20 mg of cortisol on a daily rhythm. Its highest concentration in the blood is observed in the morning, between 8 and 10 a.m. [1]. However, cortisol levels fluctuate particularly in stressful situations, but also when the body’s diurnal rhythm is dysregulated, e.g., when the sleep–wake rhythm is disrupted. Unfortunately, as scientific studies indicate, the levelling of cortisol secretion is relatively slow. In addition, glucocorticosteroids alter the expression of biological clock genes in the kidneys, lungs, and muscles, which may further slow down the synchronisation of the diurnal clock [2].

Glucocorticoid receptors are located in peripheral tissues as well as in the central nervous system [2]. Long-term stress, and consequently prolonged high cortisol concentrations, highlight the toxic effects of the hormone, not only leading to metabolic changes such as diabetes, osteoporosis, or muscle cachexia, but also changes in the central nervous system [1]. Changes in brain cortisol levels affect both behaviour and cognitive function. Initially, elevated levels of cortisol cause euphoria, but prolonged exposure of the brain to high levels of cortisol results in the appearance of other psychological changes, such as irritability, emotional labile, and depression [3]. It is also common to observe excessive cortisol secretion in about 50% of patients suffering from depression [4]. The concentration of this hormone can also be used as an indicator of drug resistance [5]. Elevated cortisol levels do not only accompany depression. It can also occur in bipolar disorder (BD), where an increase in cortisol secretion may be associated with the manic phase [6,7].

The toxic effects of cortisol can cause damage to the central nervous system [8], which can consequently impair cognitive function. Elevated cortisol levels relative to a control group have also been observed in individuals with psychotic major depression and schizophrenia spectrum disorders [9]. Some studies indicate that hypercortisolemia may lead to psychiatric disorders [8] and that the accompanying stress may lead to psychosis [10]. In addition, results from studies with psychosis sufferers show that they have significantly higher cortisol levels than controls [11,12,13]. Abnormal secretion of the hormone was particularly evident in patients not treated with antipsychotic medication [12]. Elevated levels of the hormone appear to be correlated with the risk of a first psychotic episode, in particular [13], but symptom severity is correlated with it in only the initial phase of psychosis [14,15,16], particularly with positive symptoms [14] or increased anxiety [16].

Determination of cortisol levels may be indicated not only in patients suffering from depression, during which an increase in the hormone is often observed. Additionally, in schizophrenia and BD, testing cortisol levels may indicate a blunt cortisol awakening response of the hypothalamic–pituitary–adrenal axis (HPA axis), where cortisol levels correlate with the severity of symptoms of the illness [17,18]. Furthermore, studies suggest that paranoid schizophrenic patients experience hypersensitivity of dopamine receptors in the hypothalamus, resulting in increased HPA axis activity. In contrast, in the depressive phase of BD, increased HPA axis activity is associated with thyroglobulin disturbances. These observations may explain the different stress responses in individuals affected by these conditions [19]. Stressful situations and, until recently, the ongoing pandemic and associated isolation and loneliness may also contribute to elevated cortisol levels and consequently become a reason for the emergence of depressive disorders.

Dysregulation of the HPA axis and associated changes in cortisol levels have been observed, among others, in COVID-19. It has been found that abnormal levels of the hormone can be a predictor of the severity of the progression of the disease [20]. It was found that cortisol levels can both decrease and increase during the disease course. High cortisol levels were most common in the early stages of the disease, but the severe course of the disease resulted in impaired adrenal function and consequently reduced levels of the hormone. Hence, the concept of using cortisol as a therapy in the course of COVID-19 also arose [21].

For people who have undergone COVID-19 and have not required hospitalisation, studies have suggested that the stress of surviving the disease causes chronic peripheral inflammation. This is particularly evident in people suffering from long-term COVID-19, which is a multi-organ syndrome characterized by multiple clinical manifestations from multiple organs, such as the lungs or cardiovascular system [22].

The determination of cortisol may be indicated, among other things, in psychiatric disorders, where cooperation with the patient may be difficult. The basic diagnostic material is blood, which makes it possible to determine the concentration of the analysed compounds in both free and protein-bound forms. However, blood collection is associated with numerous inconveniences, such as the obligation to use disposable, sterile equipment and the involvement of qualified people. In the case of cortisol determination, the stress of blood collection can further affect not only the patient’s discomfort but also the level of the compound being determined. In addition, for children or elderly people, the traditional determination of the hormone in the blood is difficult to perform and uncomfortable for the patient. Therefore, monitoring of cortisol levels can be performed in saliva, due to the high correlation between blood and salivary levels of the hormone [23]. Saliva as a diagnostic material allows for non-invasive sampling, which is a great advantage for those afraid of needles. In addition, saliva can be taken independently by the volunteer and does not require the presence of specialised personnel. Saliva allows the determination of concentrations of compounds that are non-protein-coupled. On the other hand, it is this form of the compound that is responsible for activity and action in the body.

In the case of saliva, the determination of cortisol is most often preceded by a sample purification step. This may involve precipitation [24], which, although a very simple method, does not always allow satisfactory results to be obtained, and numerous peaks from the biological matrix are then present on the chromatograms. Therefore, different types of extraction, both liquid–liquid and solid-phase, and their modifications are most commonly used to isolate cortisol from saliva. The isolation of cortisol from a biological matrix using liquid–liquid extraction involves the use of volatile and toxic solvents, although in the case of saliva, it gives a good purification effect from endogenous compounds and is therefore often used with this biological matrix [25,26]. A method that allows isolation with good yields and that significantly reduces the use of volatile solvents is solid-phase extraction (SPE). For cortisol, the cartridges used are those with non-polar properties Strata-X [27], HLB [28,29,30,31] and C18 [32,33].

Among the methods to determine cortisol levels in the body, both immunochemical [34,35] and separation methods are used. The most common is liquid chromatography coupled to mass spectrometry [31,36,37] or DAD detection [38]. Although MS/MS (mass spectrometry) detection is the most sensitive, the cost of its use and maintenance is very high. DAD detection also allows the determination of cortisol with sufficient sensitivity and is less expensive and, therefore, also common.

The correlation that exists between cortisol concentrations in blood and saliva allows saliva to be used as a valuable diagnostic material. In saliva, the content of the active substance is often lower than in blood, so most analytical procedures developed for their determination require the collection of a larger volume (approximately 1 mL) of biological material for study. In the case of patients using psychotropic drugs, obtaining such a volume of saliva may be difficult due to emerging side effects such as dry mouth. Taking this into account, we aimed to develop a sensitive analytical method to determine cortisol in a small volume of human saliva (200 µL). The isolation of cortisol was performed using SPE columns, thus reducing the use of volatile solvents, and the determination was performed using HPLC with DAD, further reducing the cost of the study. In addition, the developed and validated method was applied to the determination of cortisol in healthy subjects and COVID-19 patients using psychotropic drugs. In both cases, samples were taken around 9 a.m., when body cortisol concentrations are the highest.

## 2. Materials and Methods

### 2.1. Chemicals and Solvents

Hydrocortisone was purchased from Sigma-Aldrich (St. Louis, MO, USA). The internal standard (IS) chlordiazepoxide was purchased from Polfa Tarchomin (Warsaw, Poland). Methanol of HPLC gradient grade was obtained from POCh (Gliwice, Poland). Acetonitrile (Merck, Darmstadt, Germany) was HPLC super-grade and water was purified by Ultra-Toc/UV system, Hydrolab (Straszyn, Poland). For solid-phase extraction columns, Strata-X (30 mg/3 mL) were purchased from Phenomenex (Torrance, CA, USA).

### 2.2. LC-DAD Conditions

For chromatographic analysis, a Nexera XR UHPLC liquid chromatograph (Shimadzu, Kyoto, Japan) consisting of an LC-30AD pump, CTO-20AC thermostat, SIL-30AC autosampler, SPD-M30A UV-VIS detector with diode array, and SPD-M30A high-sensitivity measuring cell (85 mm), controlled by a CBM-20 Alite control system, was used. Chromatographic separation was carried out using a Eurospher II 100-3 column (C18, 50 × 4.6 mm, 3 µm; Knauer, Berlin, Germany) with precolumn maintained at 35 °C. Acetonitrile and water constituted the mobile phase at a flow rate of 0.9 mL/min, with a linear gradient with an initial acetonitrile concentration of 25% and a final concentration of 90%, which increased until 9 min. Afterwards, the acetonitrile content decreased to the initial value within 2 min. The total analysis time including column equilibration was 11 min.

### 2.3. Preparation of Quality Control (QC) and Standard Solutions

The standard solutions of hydrocortisone (1 mg/mL) and chlordiazepoxide (internal standard, IS) (1 mg/mL) were prepared by dissolving 1 mg of the substances in methanol. The stock solutions were used for preparing the calibrations and QC samples. All the working solutions were obtained by diluting stock solutions with methanol. All solutions were stored at −21 °C.

### 2.4. Collection of Saliva Samples

Saliva samples were collected with Salivettes^®^ (Sarstedt, Nümbrecht, Germany) from healthy volunteers in the late evening when cortisol level is below 1 µg/mL, i.e., undetectable to the UV detector. Before collection, volunteers did not consume food or drink for approximately 30 min. They were also required to rinse their mouths with water immediately before placing the swab in their mouths. Salivettes were placed in the mouth for approximately 2 min and centrifuged at 8000 rpm for 5 min, before the resulting saliva was frozen and stored at −21 °C until analysis.

### 2.5. Extraction Procedure

Thawed saliva samples were centrifuged. Then, 200 µL were transferred to tubes and diluted with redistilled water (1:1; *v*:*v*). To all except the blank, appropriate volumes of cortisol and internal standard were added. Samples were applied to activated columns, the sorbent of which was washed with 500 µL of methanol/water mixture (10:90; *v*:*v*) and dried for 10 min, after which analytes were eluted with methanol (500 µL).

### 2.6. Method Validation

The validation process was performed in accordance with the EMA guidelines [39].

#### 2.6.1. Linearity

The linearity of the method was determined for cortisol concentrations in the range of 4–500 ng/mL by analysing four curves taken on four consecutive days. Working solutions of cortisol were added to 200 µL of saliva so that the final cortisol concentrations were 4, 50, 150, 250, 400, and 500 ng/mL. The final internal standard (IS) concentration was 300 ng/mL. The calibration curve was determined from the relationship of the analyte peak area to IS as a function of analyte concentration.

#### 2.6.2. Lower Limit of Quantification LLOQ and Limit of Detection

The limit of detection (LOD) was determined as the lowest concentration at which the analyte signal is three times higher than the background signal. The LOD was determined empirically by analysing loaded saliva samples with decreasing cortisol concentrations. Each concentration tested was analysed five times.

LLOQ was defined as the lowest concentration that could be quantified with adequate precision (coefficient of variation (CV) < 20%) and accuracy (target concentration ± 20%). LLOQ was evaluated by analyzing five replicates.

#### 2.6.3. Accuracy and Precision

According to EMA guidelines, precision and accuracy were determined by analysing the calibration curve on one day (within a day) and on consecutive days (between days). Within-day precision and accuracy were determined for four cortisol concentrations (LLOQ, low QC, medium QC, and high QC) by analysing five replicates at each QC concentration level in each analytical run. Accuracy and precision between days were assessed by analyzing each QC concentration level in 5 analytical series over 3 days. The method was considered accurate when it was ±15% of the nominal concentration at each concentration level, except for LLOQ which should be ±20%.

#### 2.6.4. Selectivity

In accordance with EMA guidelines for analytes that may be endogenous compounds, the selectivity of the method was determined from chromatograms of extracts of 10 saliva samples taken in the late evening when cortisol concentrations are below the designated detection and quantification limit (<1 ng/mL).

#### 2.6.5. Absolute Recovery and Extraction Recovery

Extraction efficiency and absolute recovery (method performance) were determined for four cortisol concentrations (LLOQ, low, medium, and high QC) and an internal standard. Absolute recovery was determined by comparing the peak area of the analytes to the peak area of the standard solutions. The analysis was performed six times for each concentration. A value exceeding 50% of the mean peak area of the six pure standard solutions for a given concentration was considered an acceptable result.

The extraction efficiency was determined by comparing the peak areas of the extracted analytes with the peak areas obtained by loading the blank extracts after the extraction process. A value exceeding 50% of the mean value of the post-spiked saliva samples for each concentration was considered an acceptable result.

#### 2.6.6. Matrix Effect

The matrix effect was determined by analyzing samples with LLOQ and three QC concentrations of cortisol (4, 15, 200 and 350 ng/mL). Peak areas of 6 extracted blank post-spiked saliva samples were compared with peak areas of 6 neat standards at each concentration. The matrix effect was calculated using the equation:

100 × (post-spiked saliva samples − neat standards)/neat standards

The result was considered acceptable if the equation value did not exceed 20%.

#### 2.6.7. Stability

The stability of the analytes in the biological matrix was determined by performing a freeze–thaw test at −21 °C. The test was performed for four cortisol concentrations of 4, 15, 200 and 350 ng/mL. For this, 800 µL of saliva was pipetted into a tube and loaded with the specified amount of analyte. Then 200 µL was taken, an internal standard was added, the sample was extracted, and chromatographic analysis was performed. The remainder of the sample was frozen and analysed in the following days. Three replicates were performed for each concentration.

Analyte stability was also determined by storing the analyte extracts in an autosampler at 15 °C for 72 h. The test was performed for three concentrations of analyte, and each concentration was analysed five times.

A result was considered acceptable if the analyte concentration did not decrease by more than 15% during storage, regardless of the storage method.

### 2.7. Clinical Application

The developed method was then used to determine salivary cortisol levels in healthy subjects (n = 16), not using any drugs acting on the central nervous system, and patients of the Hospital for the Nervous and Mentally Ill in Starogard Gdanski (n = 16) who were using psychotropic drugs. The hospital patients participating in the study recovered from COVID-19 infection as confirmed by the test. They were presented in detail with the purpose of the study, signed an informed consent to participate, and were informed that they could withdraw from participation at any time during the study. The study protocol was approved by the ethical committee of the Medical University of Gdansk, Poland (NKBBN/393/2021; 25 June 2021).

Inclusion criteria for recovering patients were a history of COVID-19 confirmed by a test and the use of psychotropic medication. Patients were recruited in one hospital ward to exclude the influence of other factors, such as additional activities or a different daily schedule. For the control group, all subjects were healthy and had not undergone any infection in the month up to the time of sampling.

Participants’ saliva samples were collected once with Salivettes^®^ (Sarstedt, Nümbrecht, Germany) around 9 a.m., when salivary cortisol concentration is the highest. Samples were collected and prepared for chromatographic analysis according to the procedure described in Section 2.4 and Section 2.5.

### 2.8. Statistical Evaluation of the Results

Statistical evaluation of the results obtained was performed using Statistica (13.3). Statistically significant differences in cortisol concentrations between the different groups were determined using the Wilcoxon test, and the significance level was set at *p* < 0.05.

## 3. Results

### 3.1. Chromatographic Analysis

**Figure 1:**
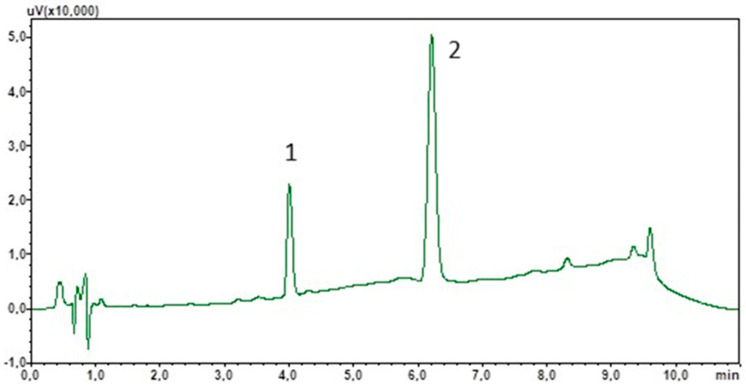
Chromatogram of the standard solutions obtained by optimised LC. (1) Cortisol; (2) chlordiazepoxide (IS).

To optimise the chromatographic separation, an appropriate mobile phase composition and flow rate were selected. Due to the non-polar properties of cortisol and IS, the stationary phase used was sorbent C18. Furthermore, due to the target use of the method for the determination of cortisol in saliva samples of varying composition, a mobile phase gradient was used. This will also allow the elution of lipophilic components from the matrix, which often interact strongly with the stationary phase. The total analysis time was 11 min and peak detection was carried out at 240 nm. Figure 1 shows a chromatogram showing the separation of the standard solutions under the optimised analysis conditions.

### 3.2. Solid Phase Extraction

The optimisation of the extraction process involved several aspects. The first was the selection of a suitable cartridge. Here, the physicochemical properties of cortisol and IS were guided, so initial tests were carried out with three types of columns differing in sorbent type (C18, HLB, Strata-X), mass, and capacity of 20 mg/1 mL, 25 mg/1 mL, and 30 mg/1 mL, respectively. Blank saliva samples and cortisol spiked samples at 15, 200, and 350 ng/mL were analysed at this stage of the study. Each type of column was activated with methanol and water, and then saliva samples diluted with water (1:1; *v*:*v*) were applied to the columns. Due to the lack of dissociation in the case of cortisol and IS, water with a small admixture of methanol (1:9; *v*:*v*) was used to wash the cartridges. The use of methanol served to remove the ballast derived from the lipophilic matrix. The next step was to dry the sorbent for 10 min, after which the analytes were washed with methanol. Due to the different masses of the sorbent used in the columns and their capacities, the volumes of the solvents used were increased to 0.5 mL for the Strata-X columns. Based on the results obtained, the Strata-X columns were used for the next stages of the study.

### 3.3. Method Validation

The linearity of the method was determined in the concentration range of 4–500 ng/mL. Detailed results are shown in Table 1. These confirmed that the method was linear and that it met the acceptance criteria. The LLOQ of cortisol was determined at 4 ng/mL and the LOD at 2 ng/mL. In addition, the determined validation parameters of the method, presented in Table 2, showed that the method was precise and accurate. For all concentrations tested, the CV for inter-day precision did not exceed 8%, while intra-day precision was 12%. The results obtained also met the EMA requirements (CV% < 15%) for LLOQ (CV% < 20). The accuracy of the method was also confirmed, as none of the concentrations tested differed from the nominal concentration by more than 1.4%.

The selectivity of the method was confirmed by analysing the chromatograms of the saliva samples taken in the late evening and comparing them with the chromatograms of the saliva extracts taken around 9 a.m. No cortisol peak was detected for the samples taken in the evening, indicating that the levels of the hormone tested in the evening were below the detection level. However, in the case of chromatograms of saliva extracts taken in the morning, a cortisol peak was present. In contrast, there were no peaks during IS retention on the chromatograms regardless of the hour of sampling.

The extraction efficiency, determined as absolute recovery and extraction recovery, was tested for four cortisol concentrations (4, 15, 200, and 350 ng/mL) and the results obtained are summarised in Table 2. In the absolute recovery test, the mean value of the peak areas of post-spiked extracts of saliva samples was compared with the mean value of the peak areas obtained from chromatographic analysis of cortisol solutions. Meanwhile, for investigating extraction recovery, the mean value of the area of cortisol peaks extracted with the developed method was compared with the peak areas of post-spiked extracts saliva samples.

According to EMA guidelines, both absolute recovery and extraction recovery should exceed 50%. For the cortisol concentrations analysed, extraction efficiency ranged between 79.11 and 86.55%. In contrast, absolute recovery for concentrations of 200 and 350 ng/mL exceeded 90%, only for the lowest cortisol QC concentration (15 ng/mL) was 80.68%. Based on the results obtained, it can be concluded that extraction recovery and absolute recovery meet the designated acceptance criterion for all concentrations tested.

The matrix effect was determined by comparing the mean area of six extracted blank post-spiked saliva samples with peak areas of six neat standards at each concentration. The results of the study are presented in Table 2. It can be concluded that for the tested concentrations the matrix effect was 0.49 and 4.71%, respectively, and this meets the requirements of the EMA (<20%).

The stability of cortisol was tested for four concentrations (4, 15, 200, and 300 ng/mL) of the analyte at 8 °C and −21 °C, and the results are shown in Table 2. The stability results are presented as a loss of concentration relative to the concentration determined on day one and are expressed as % loss. Both tests showed that cortisol was stable under the conditions tested.

### 3.4. Clinical Application

**Figure 2:**
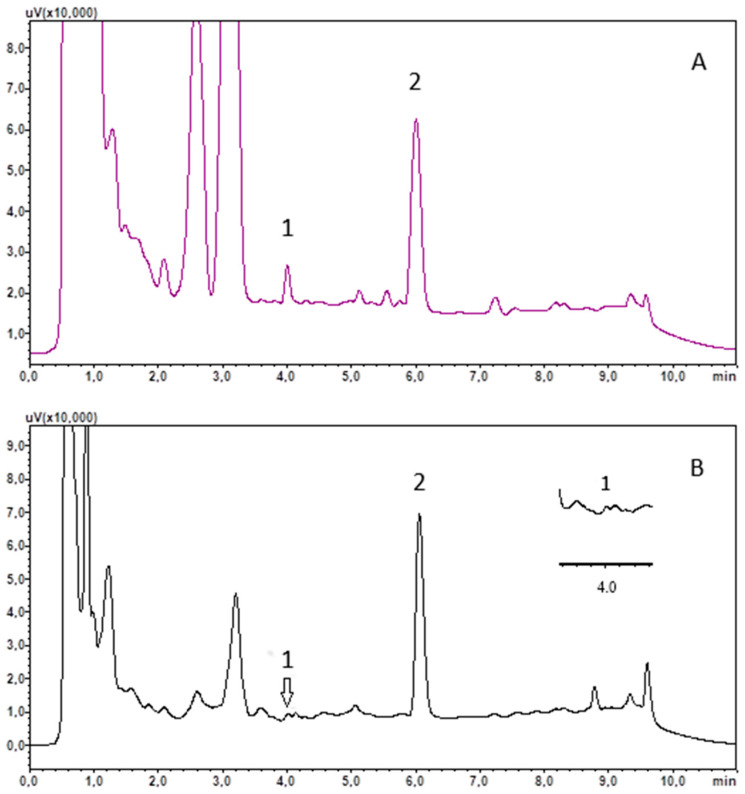
Chromatograms of saliva extract of recovery R13 (**A**) and control C8 (**B**). (1) cortisol; (2) chlordiazepoxide (IS).

The usefulness of the developed method was tested by analysing saliva samples, collected at around 9 a.m. from 16 healthy subjects and 16 COVID-19 recoveries. The saliva was treated using the procedure outlined in Section 2.4 and Section 2.5. The results obtained are included in Table 3, and example chromatograms of the saliva extract are shown in Figure 2.

### 3.5. Statistical Analysis of Results

**Figure 3:**
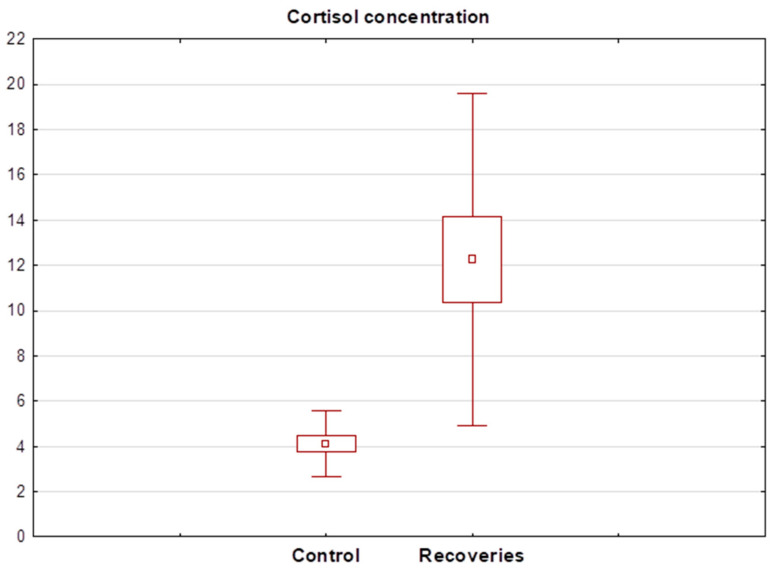
Box plot of cortisol concentration determined in the saliva of recoveries (n = 15) and healthy control group (n = 16).

Statistical analysis showed that cortisol levels in healthy subjects ranged between 2.31 and 7.14 ng/mL (mean 4.11 ± 1.46 ng/mL). In contrast, salivary levels of the hormone in the recovered patients ranged between 3.59 and 108.20 ng/mL (mean 18.24 ± 25.01 ng/mL). The Wilcoxon test showed a significant statistical difference between the mean cortisol concentration in the recovery group and healthy subjects. The cortisol concentration determined in recovery R13 was significantly different from the others and was 108 ng/mL, so the result was discarded in the next step and the Wilcoxon test was performed again. Again, this showed a statistically significant difference between the salivary cortisol concentrations of healthy and recovered individuals. A detailed summary of the determined salivary cortisol levels in each group of subjects is shown in Table 3. A box plot showing the mean cortisol concentrations determined in both groups after the exclusion of the R13 recovered patient is shown in Figure 3.

## 4. Discussion

Cortisol, known as the stress hormone, increases in numerous disease states such as during viral infection or depression. The level of cortisol due to the existing correlation can be measured in both blood and saliva. Psychotropic drug users often have a reduction in saliva secretion, so an important aspect of working with this biological material is to develop appropriate procedures that allow compounds to be determined in the smallest possible volume of saliva. The method developed allows cortisol concentrations to be measured in 200 µL of biological material. This is important when cortisol levels are being determined in people using psychotropic drugs that can cause dry mouth. In the case of our study, saliva samples were specifically collected from people receiving psychiatric treatment and using CNS-active preparations. For the literature data, the volume of saliva collected ranged between 250 and 1000 µL [24,25,26,27,28,29,30,31,33]. Only a few studies required a volume equal to or less than 200 µL [32,36,37]. However, mass spectrometry was then used to detect cortisol. However, mass spectrometry was then used to detect cortisol. Another important aspect of the developed method is that UHPLC with DAD detection, which as a type of chromatography is widely used, was applied for the determination of the hormone. Furthermore, this type of detection is cheaper to use than the frequently used MS/MS. This is important for routine monitoring of cortisol levels.

The method developed has high precision and accuracy, as evidenced by the low CV for both intra- and inter-day precision. In both cases, the CV did not exceed 8% and 12% (respectively intra and inter-day), thus meeting the EMA guidelines. Also, the extraction method developed had a high efficiency and for all concentrations analysed exceeded 79% for extraction recovery and 80% for absolute recovery. Cortisol was stable both in the saliva samples during the freeze–thaw test and when the extracts were stored in the autosampler. In both cases, analyte loss did not exceed 5% for any of the concentrations tested.

The next stage of the study was to use the developed method to determine cortisol concentrations in saliva samples of recovered patients who had been hospitalised and treated with psychotropic drugs. The results were then compared with cortisol concentrations determined in the saliva of healthy individuals. The determined cortisol levels were 12.24 ± 7.33 ng/mL in the recovered patients and 4.11 ± 1.46 ng/mL in the healthy subjects, respectively. Cortisol in the recovered patients was determined after COVID-19 infection, but this was the Omicron variant, which was less virulent than the earlier variants. Previous studies indicated that cortisol levels increased during the pandemic, which was mainly due to perceived anxiety and loneliness [40,41]. Elevated cortisol levels have also been observed in healthcare workers as a result of strain, burnout, and exhaustion [42]. Elevated cortisol levels during the prevailing pandemic may have resulted from confinement and loneliness [43,44]. However, our study aimed to determine whether simply experiencing an infection with a milder course would result in elevated levels of the hormone. Previous studies have indicated a correlation between cortisol levels with COVID-19 symptom severity [45].

Sustained high cortisol levels over a prolonged period can negatively affect the brain and consequently contribute to depressive symptoms. The recovering patients whose cortisol levels were determined were treated with psychotropic drugs, which, according to our previous studies, lower cortisol levels [46,47]. In this case, despite the use of these drugs, cortisol levels were significantly increased, which was also confirmed by statistical analysis. Some studies suggest a direct effect of the SARS-CoV-2 virus on the HPA axis, increasing hormone secretion [48]. The virus itself affects the function of the HPA axis through direct action on both the hypothalamus and adrenal glands, causing vasculitis, for example. In addition, it may reduce cholesterol levels, which is a substrate for cortisol production. Our study may also indicate that a mild history of COVID-19 infection does not protect patients from virus-related complications. In addition, the applied treatment with psychotropic compounds, which normalise cortisol levels in people with psychiatric disorders during therapy, does not protect patients from a rise in the hormone during COVID-19.

## 5. Conclusions

An effective method for the determination of cortisol in a small volume of biological material was developed and validated, which also allows the hormone level to be determined in people with salivary disorders. The method was then used for the determination of the hormone in healthy people and COVID-19-recovered patients and the results were compared. Statistical evaluation shows that cortisol levels are statistically significantly different in the two groups. It may indicate that the elevated cortisol levels were mainly influenced by the infection and that the psychotropic drugs used, which usually lower the hormone, had a negligible effect on the concentration.

## Figures and Tables

**Table 1:** Calibration curve parameters for the developed method.

Calibration Curve *y* = *ax* + *b* (n = 4)	
Range (ng/mL)	4–500
Determination coefficient (R^2^)	0.9986 ± 0.0009
Slope a ± Δa	0.0011 ± 0.0001
Intercept b ± Δb	−0.0036 ± 0.0065
LLOQ (ng/mL)	4.0
LOD (ng/mL)	2.0

**Table 2:** Validation parameters for the developed method. Stability of cortisol at LLOQ and three QC concentrations after storage in the 8 °C and −21 °C are expressed as % loss.

QC (ng/mL)	Intra-Day (CV%)	Inter-Day (CV%)	Extraction Recovery (%)	Absolute Recovery (%)	Matrix Effect (%)	Stability (Difference%)	
						8 °C	−21 °C
4	6.16	8.13	81.22	82.10	8.34	−1.28	−3.05
15	7.87	9.81	80.29	80.68	0.49	−0.47	−3.42
200	0.85	11.97	79.12	91.25	5.35	−2.52	−4.89
350	2.80	10.86	86.55	90.63	4.71	−0.21	−2.31

**Table 3:** Concentrations of cortisol found in the saliva of recoveries (n = 16) and healthy control group (n = 16).

Concentration of Cortisol (ng/mL)			
Recoveries		Control	
R1	13.33	C1	3.60
R2	17.87	C2	6.53
R3	8.35	C3	5.65
R4	5.58	C4	2.41
R5	6.12	C5	2.31
R6	14.28	C6	5.56
R7	8.78	C7	3.56
R8	8.19	C8	7.14
R9	3.59	C9	4.22
R10	25.56	C10	2.85
R11	7.34	C11	3.58
R12	18.22	C12	4.63
R13 *	108.20	C13	2.81
R14	8.41	C14	4.17
R15	9.74	C15	2.79
R16	28.27	C16	3.93
Mean ± SD	12.24 ± 7.33		4.11 ± 1.46
Median	8.78		3.765

*—as the outlier result; it was not taken into account for further statistical calculations.

## Data Availability

The original contributions presented in the study are included in the article; further inquiries can be directed to the corresponding author.

## Abbreviations

The following abbreviations are used in this manuscript:

CNS	Central Nervous System
LC	Liquid chromatography
DAD	Diode array detector
CV	Coefficient of variation
ACTH	Adrenocorticotropic hormone
CRH	corticotropin-releasing hormone
BD	Bipolar disorder
HPA axis	Hypothalamic–pituitary–adrenal axis
SPE	Solid phase extraction
MS	Mass spectrometry
IS	Internal standard
QC	Quality control
EMA	European Medicines Agency
LOD	Limit of detection
LLOQ	Lower limit of quantification
